# Comparing the Effects of Cranial Electrotherapy Stimulation and Cognitive Behavioral Therapy for Insomnia on Daily Mood and Physiological Sleep Parameters in Athletes with Poor Pre-Competition Sleep Quality

**DOI:** 10.3390/life15060905

**Published:** 2025-06-03

**Authors:** Yung-An Tsou, Bao-Lien Hung, Wen-Dien Chang

**Affiliations:** 1 Department of Otolaryngology-Head and Neck Surgery, China Medical University Hospital, Taichung 40447, Taiwan; d22052121@gmail.com; 2 Department of Audiology and Speech-Language Pathology, Asia University, Taichung 41354, Taiwan; 3 School of Medicine, China Medical University, Taichung 404328, Taiwan; 4 Department of Sports Medicine, China Medical University, Taichung 406040, Taiwan; blhoung@mail.cmu.edu.tw; 5 Department of Sport Performance, National Taiwan University of Sport, Taichung 404401, Taiwan

**Keywords:** sleep quality, heart rate variability, sleep architecture

## Abstract

This study aimed to compare 4 weeks of cranial electrotherapy stimulation (CES) versus cognitive behavioral therapy for insomnia (CBT-I) in athletes with poor sleep quality pre-competition as measured by changes in daily mood state and physiologic parameters of sleep. Athletes with poor sleep quality in their pre-competition phase were recruited. Four weeks of CES and CBT-I were used to compare the effects on daily mood state and physiologic parameters of sleep. The participants were divided into a CES and a CBT-I group. The Pittsburgh Sleep Quality Index (PSQI), Epworth Sleepiness Scale (ESS), Profile of Mood States (POMS), nighttime heart rate variability (HRV), and sleep architecture of cardiopulmonary coupling (CPC) analyses were assessed before and after the interventions. Twenty-four participants (time to competition = 46.71 ± 11.21 days) completed the study. Decreases in PSQI and ESS scores were observed in both groups. A decrease in confusion and tension scores and improvement of sleep efficiency were noted after CBT-I (*p* < 0.05). Changes in light sleep (stages S1 and S2) and deep sleep (stages S3 and S4) were observed (*p* < 0.05), accompanied by alterations in HRV (*p* < 0.05). Both interventions for athletes experiencing poor sleep quality before competition had efficacy in improving sleep quality and reducing daytime sleepiness. CES could cause alterations in sleep architecture and autonomic nervous regulation, and CBT-I contributed to a reduction in negative mood states. This study is tiny and limited by the absence of a control group, which may introduce psychological bias, and future research should include control conditions and extended follow-up assessments to validate these findings.

## 1. Introduction

Adequate sleep is essential for athletic performance, recovery, and overall well-being. However, many athletes suffer from sleep disturbances before competitions, often due to heightened stress, anxiety, and disruptions in routine [1]. Pre-competition sleep disturbances are characterized by reduced total sleep time, decreased sleep efficiency, increased waking after sleep onset, and poorer sleep efficiency [2]. There is a connection between physiological recovery during sleep and training efficiency and sport performance in athletes. Overtraining syndrome or chronic fatigue is a prevalent issue that negatively impacts athletes, contributing to sleep debt, which primarily affects overall health and sport performance [3]. A previous study indicates that even a single night of poor sleep before competition could affect reaction time, decision-making, and endurance performance, increasing the risk of sport injury and suboptimal performance outcomes [4].

Cranial electrotherapy stimulation (CES) could be a non-pharmacological intervention for addressing sleep disturbances in athletes, who frequently experience sleep problems due to pre-competition anxiety. By delivering low-intensity electrical currents, CES modulates brain activity, enhances parasympathetic function, and regulates neurotransmitter release, which are essential for sleep regulation [5,6]. Empirical studies demonstrated the efficacy of CES in reducing sleep onset latency, improving sleep efficiency, and stabilizing sleep architecture, particularly in high-performance populations such as military personnel and individuals with stress-induced insomnia [7,8]. Given that poor sleep quality negatively impacts cognitive function, physiological recovery, and injury risk in athletes, CES may serve as a novel tool for optimizing pre-competition sleep, enhancing post-exercise recovery, and managing circadian rhythm disruptions. With its non-invasive nature and demonstrated usage in improving sleep in a specific population, CES may hold potential for integration into athlete recovery protocols, further research is needed to discuss its application in practice settings.

Recognized by the American Academy of Sleep Medicine (AASM), cognitive behavioral therapy for insomnia (CBT-I) incorporates multiple strategies to address sleep behaviors and sleep disturbances [9]. CBT-I includes components such as sleep restriction, stimulus control, cognitive restructuring, relaxation techniques, and sleep hygiene education, targeting the behavioral and cognitive factors contributing to poor sleep. Several studies demonstrated that CBT-I had effects on reducing sleep onset latency, improving sleep efficiency, and enhancing overall sleep quality among athletes, ultimately leading to better recovery, mood regulation, and cognitive function [10,11]. For athletes, optimal sleep is essential for peak performance, recovery, and sport injury prevention; therefore, effective interventions for poor sleep quality in the pre-competition period are needed. Therefore, the primary aim of this study was to compare the effects of a 4-week intervention using CES versus CBT-I on pre-competition sleep quality in athletes. We hypothesized that both interventions would improve daily mood state and physiologic parameters of sleep, with differential effects between the two interventions.

## 2. Methods

### 2.1. Study Procedures

This study was a randomized control study. We recruited college athletes of combat sports from the sports teams of sports university. The inclusion criteria required the participants to be within 2 months of a national-level competition, have experienced insomnia before a competition, and have a single-item sleep quality scale (SISQ) score of less than 3. The SISQ is a subjective measure that assesses overall sleep quality over one week, rated on a scale from 0 to 10, where 0 represents extremely poor sleep quality and 10 represents excellent sleep quality [12]. The exclusion criteria included individuals with a history of epilepsy, those prone to dizziness induced by physical activity, individuals with a pacemaker, pregnant participants, and those unable to complete the experimental procedures. Twenty-four athletes were recruited and participated in our study. This study was approved by the Institutional Review Board of the China Medical University Hospital (No. CMUH108-REC3-165), and all volunteers were informed of the study procedure and agreed to participate. 

**Figure 1:**
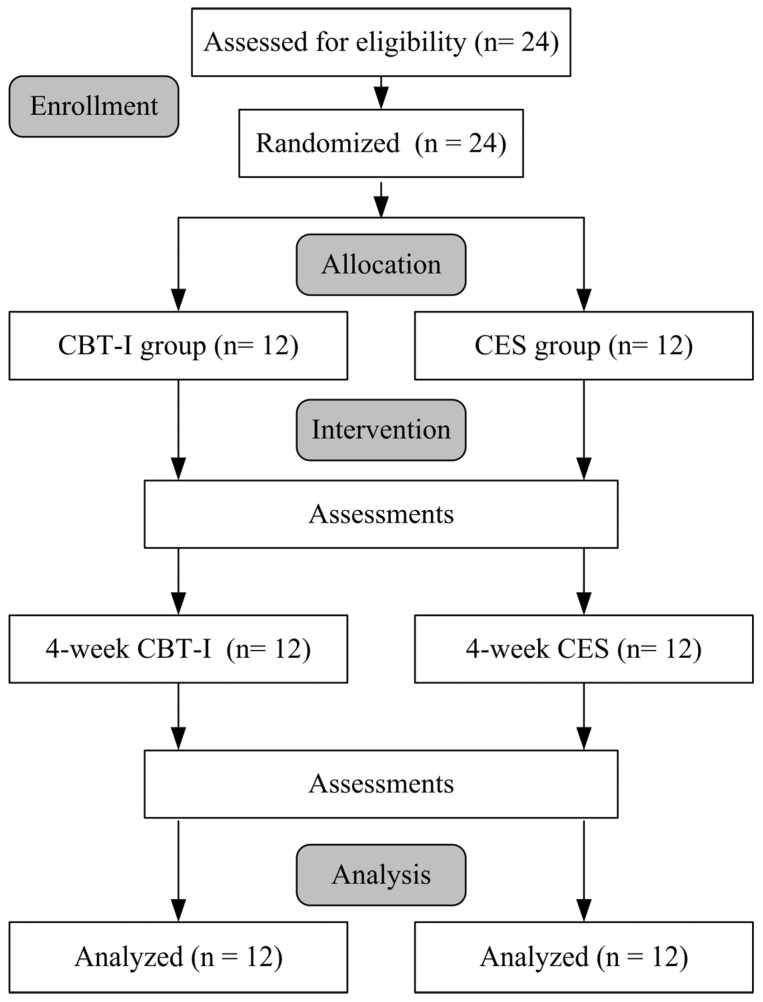
Subject testing process.

The study procedure is presented in Figure 1. All participants were randomly assigned to two groups in a 1:1 ratio using the RANDBETWEEN function in Microsoft Excel. Random numbers were generated for each participant, and the list was then sorted in ascending order. The first half of the participants were assigned to the CBT-I group, and the remaining half to the CES group. The group allocation was blinded. The Pittsburgh Sleep Quality Index (PSQI), Epworth Sleepiness Scale (ESS), profile of mood states (POMS), and cardiopulmonary coupling (CPC) analysis were assessed before and after the interventions. All assessments were conducted by a researcher, while data analysis was performed by a separate analyst in the university laboratory. 

### 2.2. Interventions

#### 2.2.1. Cranial Electrotherapy Stimulation

The Alpha-Stim microcurrent stimulator (Alpha-Stim, Electromedical Products International Inc., Mineral Wells, TX, USA) was used. The electrical parameters of 60 min, bipolar asymmetric rectangular waveform, 0.5 Hz, 100 µA, and 50% duty cycle were used. Lande and Gragnani found that administering CES at 100 μA and 0.5 Hz for 60 min over 5 days improved sleep quality and reduced sleep onset latency in male participants [13]. Therefore, the same settings and parameters were used in this study. The electrodes were placed on both earlobes for the procedure. The intervention was administered 3 times per week for 4 weeks and conducted by an otolaryngologist.

#### 2.2.2. Cognitive Behavioral Therapy

The intervention consisted of a six-session CBT-I program delivered three times per week over a period of 4 weeks. The sessions were conducted by the same psychological counselor trained in CBT-I and were supported by printed handouts containing session information [14]. The session checklist was completed after each session to ensure adherence to the protocol. The first week focused on psychoeducation regarding sleep problems, the principles of sleep hygiene, and recommended behavior changes. In the second week, participants were educated about the etiology and maintenance of insomnia and trained on how to properly complete sleep diaries. The third week concentrated on sleep restriction, providing participants with personalized sleep schedules based on the data from their sleep diaries. The fourth week addressed sleep expansion, reinforced sleep hygiene principles, and specifically focused on managing sleep problems prior to competition. 

### 2.3. Assessments

#### 2.3.1. Pittsburgh Sleep Quality Index

The PSQI consists of 7 components, including subjective sleep quality, sleep latency, sleep duration, sleep efficiency, sleep disturbances, use of sleep medication, and daytime dysfunction. The questionnaire includes 19 self-rated items and 5 additional questions rated by a bed partner or roommate. Each component is scored from 0 to 3, with a total score ranging from 0 to 21. A total PSQI score greater than 5 is typically considered indicative of poor sleep quality, and it had good reliability (reliability coefficient of 0.82–0.83) [15].

#### 2.3.2. Epworth Sleepiness Scale

ESS is a self-report questionnaire to assess the likelihood of falling asleep in 8 common daily situations. Each question is scored from 0 to 3 (0 = never, 1 = slight chance, 2 = moderate chance, and 3 = high chance), and total scores range from 0 to 24. ESS had an acceptable internal consistency (Cronbach’s alpha = 0.81) for assessing daytime sleepiness [16].

#### 2.3.3. Profile of Mood States

The Chinese version of the POMS scale was used to assess mood and emotional states. It consists of 37 items across seven distinct mood dimensions: confusion (7 items), fatigue (6 items), anger (6 items), tension (4 items), depression (3 items), vigor (7 items), and esteem (4 items). Participants rate how much they have experienced each feeling or mood during the pre-competition period using a 5-point Likert scale ranging from 1 (not at all) to 5 (extremely). The scores for each category were summed to generate a profile of the individual’s current mood. Confirmatory factor analysis indicated that the Chinese version of POMS demonstrated an acceptable fit (n = 539, χ^2^ = 167.89, *p* = 0.14) [17]. The scale exhibits appropriate construct validity and can be effectively used to assess mental attitude in sports contexts.

#### 2.3.4. Cardiopulmonary Coupling Analysis

Participants were asked to use a portable CPC device (Largan Health AI-Tech Co., Taipei, Taiwan) at home before bedtime and sleep with it on for one night. The CPC device was applied using adhesive ECG sensors placed on the chest in a standard single-lead configuration. The main unit was attached to the torso or worn near the chest area to continuously record cardiopulmonary activity during sleep. Physiological signals were transmitted via a wireless network to a cloud-based database for algorithmic processing. CPC analysis also has been developed and applied to measure the function of the autonomic nervous system using nocturnal heart rate variability (HRV) and, combined with electrocardiography signals and respiratory signals, coupled into a sleep spectrogram. By capturing and monitoring heart rate and respiratory signals, the technology can analyze the HRV and respiratory states during sleep, and further analyze sleep structure, sleep diaries, and identify stable sleep. In addition, unstable sleep can be recorded by sleep time, and the reasons for obstructive or central respiratory events affecting sleep can be analyzed [18]. In the sleep spectrogram of CPC analysis, physiologically stable sleep is associated with high-frequency (HF) coupling between heart rate and respiration at frequencies of 0.1 to 0.4 Hz. In contrast, physiologically unstable sleep is associated with low-frequency (LF) coupling between heart rate and respiration over a range of 0.01 to 0.1 Hz [19]. The autonomic nervous system plays a regulatory role in the sleep cycle. HRV is also often used in non-invasive tests to evaluate autonomic nervous function. Common signal processing methods are time domain analysis and frequency domain analysis. Time domain analysis uses electrocardiograms to record electrocardiography signals and calculate the R-R distance’s standard deviation through numerical analysis by calculating the difference of R-R distance in heart rate, which is a predictor of HRV often used in clinical practice [20]. Frequency domain analysis is used to calculate the electrocardiography signal through Fourier transform. The main frequency domain indicates that a high frequency indicates the parasympathetic nerve activity index, LF indicates the sympathetic nerve activity index, and LF/HF ratio indicates the sympathetic and parasympathetic nerve balance index [20]. 

### 2.4. Statistical Analysis

Data analysis was performed using SPSS (version 25 for Windows; SPSS Inc., Chicago, IL, USA). Data are presented as mean ± standard deviation. The Kolmogorov–Smirnov test was used to assess the normality of data distribution. An independent *t*-test was applied to compare the demographic characteristics between the two groups. A *t*-test and paired *t*-test were conducted to compare the effects within and between groups for the measured variables. A *p*-value of < 0.05 was considered statistically significant.

## 3. Results

Twenty-four participants (age = 20.63 ± 2.43 years, height = 169.73 ± 8.54 cm, weight = 63.47 ± 11.40 kg) completed the study, and there were no dropouts. As the competition approached (time to competition = 46.71 ± 11.21 days), sleep quality worsened (SISQ = 2.46 ± 0.66; insomnia frequency = 2.90 ± 1.34 times per week). Demographic characteristics, including basic data and sleep conditions, showed no significant differences between the CBT-I and CES groups (Table 1, *p* > 0.05).

Differences in POMS scores between the two groups were not significant before the intervention, indicating equivalency (Table 2). In the CBT-I group, confusion and tension scores significantly decreased after the intervention (*p* < 0.05). However, no significant differences were found in other POMS items within the CES group (*p* > 0.05). Additionally, anger, tension, and depression scores in the CES group were significantly higher than those in the CBT-I group (*p* < 0.05).

Differences in PSQI and ESS scores between the two groups were not significant before or after the intervention (*p* > 0.05, Table 3). However, significant decreases in PSQI and ESS scores were observed after the intervention (*p* < 0.05). Regarding sleep architecture, no significant differences were found between the two groups before or after the intervention (*p* > 0.05). However, a significant increase in sleep efficiency was noted in the CBT-I group, while changes in light sleep (stages S1 and S2) and deep sleep (stages S3 and S4) were observed in the CES group (*p* < 0.05).

**Figure 2:**
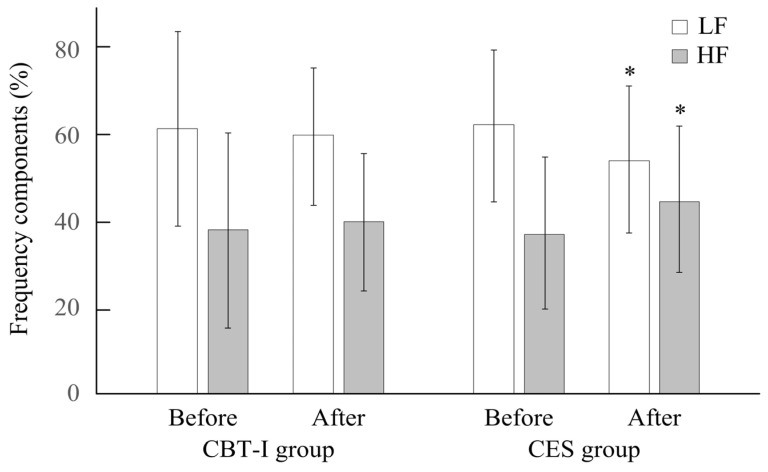
Changes in LF and HF in both groups. * *p* < 0.05, before vs. after.

For nighttime HRV in Table 3 and Figure 2, significant changes in LF, HF, and the LF/HF ratio were observed in the CES group (*p* < 0.05). Additionally, no significant differences were noted between the two groups before or after the intervention (*p* > 0.05).

## 4. Discussion

When comparing the effects of CES and CBT-I, both interventions significantly improved nighttime sleep quality and reduced daytime sleepiness. The anxiety associated with approaching competitions indirectly affected daytime psychological and sleep states. Notably, CES showed physical benefits, while CBT-I demonstrated psychological effects. This study found that after receiving CES, the percentage of deep sleep (stages S3 and S4) in sleep architecture increased. Based on the positive outcomes of PSQI and ESS, the improvement in sleep quality and daytime sleepiness with CES may be attributed to the physiological benefits of microcurrent neuromodulation, as reflected in changes in sleep architecture and HRV. In contrast, CBT-I is a psychological intervention for chronic insomnia that focuses on modifying maladaptive thoughts and behaviors related to sleep [21]. The negative moods of confusion and tension decreased after CBT-I, resulting in increase of sleep efficiency.

CES is a non-invasive device that has been used in interventions for mental and psychological issues, including insomnia, depression, anxiety, addiction withdrawal symptoms, and cognitive impairment [8,22]. Bang et al. used CES to treat chronic sleep disorders, finding significant improvements in PSQI scores (Cohen’s d = 0.40, *p* < 0.001), Insomnia Severity Index scores (Cohen’s d = 0.26, *p* = 0.006), and daily sleep logs, including sleep latency, time in bed, and total sleep time (Cohen’s d = 0.30–0.57, *p* < 0.05) [23]. In our study, CES led to improvements in PSQI and ESS, demonstrating positive effects on sleep quality and daytime sleepiness, similar to the results observed with CBT-I. Tan et al. conducted CES for poor sleep quality in athletes, and found that sleep efficiency, total sleep time, and wakefulness after sleep onset were significant improved (*p* < 0.05) [6]. The proposed mechanism behind these benefits is that CES may regulate essential neurotransmitters involved in sleep regulation [8,13,24]. Previous studies indicated that CES may influence multiple cortical and subcortical regions, including the thalamus, causing the interaction. The mechanistic effects led to neurochemical alterations, the deactivation of certain cortical areas, and the modulation of brain rhythms, which may play a role in enhancing sleep function [8,23]. However, the precise physiological, neurochemical, and metabolic mechanisms underlying CES remain to be fully elucidated.

In a study by Weiss, electrosleep treatment was used for 30 min over 10 consecutive days for insomnia, and it was found that participants fell asleep faster and woke up less frequently during the night. Electroencephalographic results showed that participants spent more time in S4 of NREM, the deepest and most restful stage of sleep [25]. Deep sleep enhanced recovery of athletes by promoting growth hormone release, supporting anabolism, and improving performance, especially following intense physical exertion [26]. One of physiological effects of CES is that it may modulate sleep architecture, particularly by increasing the proportion of deep sleep. A previous study found that, after a 2-week CES, there was an increase in the HF components of HRV, indicative of enhanced parasympathetic nervous system activity, which may positively influence autonomic function and psychological responses in athletes under pre-competition stress [27]. As sleep transitions into slow-wave sleep, it is accompanied by increased parasympathetic activity. Enhancing parasympathetic activation may therefore contribute to the strengthening of slow-wave sleep [28]. Autonomic nervous regulation is associated with sleep architecture, which is reflected in the outcomes of CES.

CBT-I commonly included stimulus control, sleep restriction, cognitive restructuring, and relaxation techniques. Research has demonstrated that CBT-I significantly improves sleep latency, reduces nighttime awakenings, and enhances sleep efficiency, with long-term benefits extending beyond the treatment period [10,29]. Unlike CES, which acts on the central nervous system, CBT-I works by altering cognitive and behavioral factors that contribute to poor sleep, making it particularly effective for individuals with persistent sleep difficulties, anxiety-related insomnia, and poor sleep hygiene. Given the high prevalence of sleep disturbances due to training schedules, competition anxiety, and travel-related disruptions [30], CBT-I could be particularly beneficial for optimizing recovery, cognitive function, and athletic performance. This intervention could stimulus control, sleep restriction, cognitive restructuring, and relaxation techniques, helping athletes establish consistent sleep patterns and reduce pre-competition anxiety [30,31]. A meta-analysis found medium to large effect sizes for improvements in sleep onset latency, sleep efficiency, and waking after sleep onset, indicating the effectiveness of CBT-I in enhancing various sleep parameters [32]. Furthermore, the negative effects of late-night training and travel-related circadian disruptions were decreased by reinforcing better sleep hygiene and psychological coping strategies for athletes [33].

A previous study indicated that 64.0% of athletes reported worse sleep on at least one occasion during the nights prior to an important competition over the past 12 months [34]. The primary difficulty reported was an inability to initiate sleep, largely attributed to pre-competition anxiety and excessive mental imagery related to performance scenarios. While the impact of sleep disturbances may vary between individual and team sports, such issues frequently arise in the pre-competition phase and, if persistent, may contribute to chronic sleep disorders [33,34]. A systematic review and meta-analysis revealed the effects of CBT-I across 15 randomized controlled trials involving 2392 individuals with insomnia. They indicated that CBT-I significantly improved sleep efficiency by 7.2%, increased total sleep time by about 20 min, and reduced both insomnia and comorbid depressive symptoms. Notably, these benefits were maintained for 4 to 28 weeks post-intervention [35]. Another systematic review identifies cognitive arousal as a key mechanism underlying insomnia and other sleep disturbances [36]. Disruptions in cognitive regulation prior to sleep could lead to intrusive thoughts and hyperarousal, precipitating sleep-onset difficulties. Additionally, attentional biases resulting from prolonged focus on intensive training may further exacerbate sleep-related issues by impairing cognitive flexibility [11,34,36]. Emotional regulation is mediated by the limbic system, which functions as a primary center for emotion processing. Sleep disturbances may trigger psychological disequilibrium and emotional dysregulation in athletes, further exacerbating pre-competition stress. By targeting maladaptive cognitive and behavioral patterns, CBT-I facilitates neurophysiological and psychological adaptations, thereby promoting improved sleep outcomes.

Due to the adverse effects associated with pharmacological treatments for insomnia, there is an increasing demand for alternative therapeutic approaches among individuals with sleep disorders. We investigated the effects of CES versus CBT-I on athletes experiencing poor sleep quality in the pre-competition phase. Studies in this area remain limited, and only one study found has evaluated the cost-effectiveness of CES and CBT-I, though specifically in individuals with generalized anxiety disorder [37]. This study highlighted critical barriers, including the shortage of trained professionals and prolonged wait times for treatment, which often limit patients’ access to CBT-I. Consequently, CES has been proposed as a viable alternative, demonstrating significant efficacy in alleviating anxiety symptoms both immediately post-treatment and at a 3-month follow-up [37]. Additionally, CES could reduce the financial burden associated with CBT-I, suggesting its potential as a comparable and substitutable intervention in experimental designs. Furthermore, a previous study explored the application of CES in behavioral regulation, demonstrating its beneficial effects in children and adolescents [24]. The findings suggested that CES may serve as a valuable adjunctive therapy by facilitating adaptation to environmental changes and modulating anxiety responses. Future research is suggested to explore the feasibility and efficacy of integrating CBT-I with CES for sleep management in athletes, particularly in addressing pre-competition sleep disturbances.

This study has several limitations. First, this study utilized a CPC device for sleep assessment due to its practicality in home settings. However, as the device has not been validated against polysomnography in current study, the accuracy of sleep stage estimation remains limited. Additionally, the absence of controls for first-night effects may have affected data reliability. Second, the limited and small sample size may introduce potential biases from self-reported measures, and the absence of a placebo or control group may increase the risk of psychological effects. This study focused on student athletes, and future research could include other populations to explore translation to real-world individuals, and larger samples are warranted to validate these preliminary findings. Third, the lack of long-term follow-up restricts the ability to assess the durability of the effects, including its prolonged benefits and potential impact on competition performance and results. Future studies should incorporate control groups and longer follow-up periods to confirm these findings.

## 5. Conclusions

In a comparison between 4-week CES and CBT-I interventions for athletes experiencing poor sleep quality before competition, both approaches demonstrated efficacy in improving sleep quality and reducing daytime sleepiness. CES primarily targets neurophysiological modulation through low-intensity electrical currents, leading to alterations in sleep architecture and autonomic nervous regulation. In contrast, CBT-I focuses on modifying behavioral and cognitive patterns that disrupt sleep, contributing to a reduction in negative mood states. Future research should incorporate control conditions and extended follow-up assessments to provide a more comprehensive evaluation of the long-term effects and potential performance benefits of these interventions.

## Figures and Tables

**Table 1:** Demographic characteristics.

	CBT-I Group (n = 12)	CES Group (n = 12)	*p*
Age (years)	20.92 ± 3.37	20.33 ± 0.89	0.57
Height (cm)	169.79 ± 8.76	169.66 ± 8.71	0.97
Weight (kg)	66.93 ± 14.13	60.02 ± 6.78	0.15
BMI (kg/m^2^)	23.11 ± 3.90	20.85 ± 1.84	0.09
SISQ	2.33 ± 0.78	2.58 ± 0.51	0.37
Insomnia frequency (times/a week)	2.83 ± 1.35	2.96 ± 1.39	0.83

* *p* < 0.05, BMI, body mass index; SISQ, single-item sleep quality scale.

**Table 2:** Outcome of POMS.

	CBT-I Group (n = 12)			CES Group (n = 12)		
	Before	After	*p*	Before	After	*p*
Confusion	15.33 ± 5.07	11.58 ± 4.85	0.01 *	18.83 ± 7.38	17.33 ± 8.75	0.11
Fatigue	10.83 ± 2.66	10.42 ± 3.18	0.69	15.67 ± 7.63	13.33 ± 5.99	0.14
Anger	7.08 ± 2.31	6.50 ± 0.67	0.29	10.01 ± 5.72	9.83 ± 4.78 ^#^	0.80
Tension	10.75 ± 2.56	8.02 ± 2.66	0.01 *	12.08 ± 3.37	11.01 ± 3.93 ^#^	0.11
Depression	3.83 ± 1.40	3.58 ± 1.24	0.27	5.58 ± 2.47	5.50 ± 2.50 ^#^	0.88
Vigor	20.50 ± 6.84	22.50 ± 7.40	0.10	18.01 ± 5.36	18.75 ± 5.46	0.35
Esteem	12.50 ± 3.37	13.17 ± 4.15	0.34	11.25 ± 3.89	11.67 ± 3.75	0.49

* *p* < 0.05, before vs. after; ^#^ *p* < 0.05, CBT-I vs. CES group.

**Table 3:** Outcome of PSQI, ESS, sleep architecture, sleep diary, and HRV.

	CBT-I Group (n = 12)			CES Group (n = 12)		
	Before	After	*p*	Before	After	*p*
PSQI	9.33 ± 2.74	6.58 ± 2.54	0.01 *	10.69 ± 3.31	6.92 ± 3.34	0.01 *
ESS	14.75 ± 4.71	10.92 ± 4.76	0.01 *	14.58 ± 3.96	12.00 ± 5.41	0.04 *
Sleep architecture						
TST (min)	407.58 ± 99.92	375.58 ± 99.01	0.42	372.33 ± 60.55	407.17 ± 77.11	0.19
Sleep efficiency (%)	86.71 ± 8.76	91.15 ± 5.15	0.03 *	89.06 ± 0.06	89.08 ± 6.50	0.91
S1 and S2 (min)	156.01 ± 61.97	159.92 ± 83.72	0.85	181.08 ± 53.59	128.08 ± 49.94	0.02 *
Percentage of S1 and S2 (%)	38.21 ± 15.33	39.86 ± 16.09	0.65	42.19 ± 7.45	32.45 ± 10.82	0.01 *
S3 and S4 (min)	155.75 ± 99.47	131.58 ± 57.14	0.33	127.42 ± 45.14	169.92 ± 64.71	0.09
Percentage of S3 and S4 (%)	34.83 ± 17.89	34.18 ± 14.74	0.85	30.63 ± 11.62	44.05 ± 15.95	0.02 *
REM (min)	84.08 ± 42.59	95.50 ± 37.84	0.39	74.33 ± 43.83	98.67 ± 26.68	0.13
Percentage of REM (%)	21.02 ± 7.89	22.25 ± 6.27	0.62	18.93 ± 9.98	23.13 ± 4.44	0.22
Nighttime HRV						
SDNN(ms)	97.50 ± 39.75	99.67 ± 27.59	0.10	99.83 ± 54.15	127.17 ± 46.78	0.06
LF/HF ratio	2.67 ± 2.36	1.90 ± 1.28	0.40	2.24 ± 1.42	1.45 ± 0.83	0.04 *

* *p* < 0.05, before vs. after; TST, Total sleep time; REM, Rapid Eye Movement.

## Data Availability

Data is contained within the article.

## Abbreviations

CES, cranial electrotherapy stimulation; CBT-I, cognitive behavioral therapy for insomnia; PSQI, Pittsburgh Sleep Quality Index; ESS, Epworth Sleepiness Scale; POMS, profile of mood states; HRV, heart rate variability; AASM, American Academy of Sleep Medicine; SISQ, single-item sleep quality scale; CPC, cardiopulmonary coupling; HF, high-frequency; LF, low-frequency.
